# An extended discrete element method for the estimation of contact pressure at the ankle joint during stance phase

**DOI:** 10.1177/0954411920905434

**Published:** 2020-02-08

**Authors:** Ivan Benemerito, Luca Modenese, Erica Montefiori, Claudia Mazzà, Marco Viceconti, Damien Lacroix, Lingzhong Guo

**Affiliations:** 1 INSIGNEO Institute for *in silico* Medicine, The University of Sheffield, Sheffield, UK; 2 Department of Automatic Control and Systems Engineering, The University of Sheffield, Sheffield, UK; 3 Department of Civil and Environmental Engineering, Imperial College London, London, UK; 4 Department of Mechanical Engineering, The University of Sheffield, Sheffield, UK; 5 Department of Industrial Engineering, Alma Mater Studiorum – University of Bologna, Bologna, Italy; 6 Laboratorio di Tecnologia Medica, IRCCS Istituto Ortopedico Rizzoli, Bologna, Italy

**Keywords:** Extended discrete element analysis, ankle, contact pressure, subject specific, nonlinear stiffness

## Abstract

Abnormalities in the ankle contact pressure are related to the onset of osteoarthritis. In vivo measurements are not possible with currently available techniques, so computational methods such as the finite element analysis (FEA) are often used instead. The discrete element method (DEM), a computationally efficient alternative to time-consuming FEA, has also been used to predict the joint contact pressure. It describes the articular cartilage as a bed of independent springs, assuming a linearly elastic behaviour and absence of relative motion between the bones. In this study, we present the extended DEM (EDEM) which is able to track the motion of talus over time. The method was used, with input data from a subject-specific musculoskeletal model, to predict the contact pressure in the ankle joint during gait. Results from EDEM were also compared with outputs from conventional DEM. Predicted values of contact area were larger in EDEM than they were in DEM (4.67 and 4.18 cm^2^, respectively). Peak values of contact pressure, attained at the toe-off, were 7.3 MPa for EDEM and 6.92 MPa for DEM. Values predicted from EDEM fell well within the ranges reported in the literature. Overall, the motion of the talus had more effect on the extension and shape of the pressure distribution than it had on the magnitude of the pressure. The results indicated that EDEM is a valid methodology for the prediction of ankle contact pressure during daily activities.

## Introduction

The determination of contact patterns of cartilage in the ankle can give an insight on the physiological behaviour of the joint.^1^ Also, abnormalities in such patterns have been linked to the onset of osteoarthritis,^2^ making therefore the investigation of the contact characteristics of the ankle of paramount importance. Early attempts of measuring the contact features of the ankle date back to cadaveric studies in the 1970s^3,4^ and since then many different methodologies have been developed for the ex vivo investigation of the contact characteristics of the joint.^5–9^ However, results from cadaveric studies were obtained in conditions substantially different from the in vivo scenarios they were trying to mimic. Likewise, the few studies estimating the ankle joint cartilage deformation in vivo^10,11^ were conducted either under constant loads or simulated mid-stance phase of walking. The difficulties in estimating the ankle contact pressure experimentally can be partially overcome by the use of in silico models which simulate the contact of cartilage layers within the joints. Finite Element Analysis (FEA) has been widely used for the investigation of the joint contact pressure on subject-specific geometries of different joints, and researchers have tested and validated its predictions at the hip,^12,13^ at the knee,^14^ and at the ankle.^15^ Hyperelastic^16^ or multiphasic^17^ descriptions of the cartilage can be treated within the framework of FEA. Numerical convergence problems and long computational time are however a common obstacle and they can be exacerbated when dealing with nonlinear materials and complex geometries.^18^ A computationally efficient alternative to FEA is the Discrete Element Method (DEM),^19–21^ which represents the bones as rigid bodies and the articular cartilage as a bed of linear elastic springs. Various studies have assessed its accuracy against FEA predictions^22–24^ and experimental results^18,25,26^ on different joints and found good agreement between corresponding predictions.

DEM has been used to predict the joint contact pressure, limiting the analysis to single independent time points of the gait cycle^23,24,27,28^ and assuming that the relative distance between the contacting bodies does not change over time, meaning that they are never displaced from their initial position. However, this is in contrast to experimental evidences obtained through intra-cortical bone-pins,^29,30^ skin reflective markers^31^ and in vivo magnetic resonance imaging (MRI),^32,33^ showing that the distance between the articulating bones varies during the gait cycle. Also, it has been shown that material properties such as the stiffness of the cartilage can change according to its strain state during a deformation process.^34–36^

The aim of the present work is to extend the classical DEM and to endow it with the capability of tracking the relative position of the contacting bodies over time, introducing the strain dependent stiffness within the modelling process. This will permit to account for the nonlinear behaviour of the articular cartilage, therefore increasing the veracity of the model.

The developed algorithm is applied, in conjunction with subject-specific musculoskeletal (MSK) modelling approach, to the prediction of joint contact pressure and joint contact area during the stance phase of the gait cycle in a subject-specific model of the ankle. Results from sensitivity analysis on EDEM (Extended DEM) inputs are also presented, and the difference in the predictions between EDEM and DEM is discussed.

## Materials and methods

### Data acquisition and MSK modelling

Gait analysis data and MRI scans were collected from one female participant (age: 16 years, weight: 68 kg, height: 160 cm) at the Istituto Giannina Gaslini (Genoa, Italy). Written informed consent was obtained from the subject and from her parents. The study was approved by the local medical ethics committees of the participating centre and conducted according to good clinical practice guidelines and the Declaration of Helsinki. The subject performed one walking trial at self-selected speed. Gait data, namely ground reaction forces and markers trajectories, were collected using two force plates (AMTI OR6-6; 1000 Hz) and a stereo-photogrammetric system (Vicon Motion System Ltd, Oxford, UK; 200 Hz) respectively. The adopted marker protocol was based on the Vicon PlugIn gait protocol (Vicon Motion System, Ltd, Oxford, UK) and the modified Oxford Foot Model.^37^ MRI scans of the lower limbs were acquired in supine position with multi-slice multi-echo three-dimensional (3D) gradient echo, with 1-mm slice thickness, 0.5-mm inter-slice gap and 0.5-mm in-plane resolution. Segmented bone geometries were imported into MeshLab^38^ to identify the articular surfaces of the articulating bones. The ankle joint, or tibiotalar joint, was modelled as an ideal joint whose axis was identified as the axis of the least-square cylinder fitted to the articular surface of the talus.^39^ This representation allowed for a description of the dorsiflexion–plantarflexion movement of the joint. Relevant reference systems were defined using proximal and distal anatomical coordinate frames according to the recommendations of the International Society of Biomechanics.^40^

**Figure 1.**
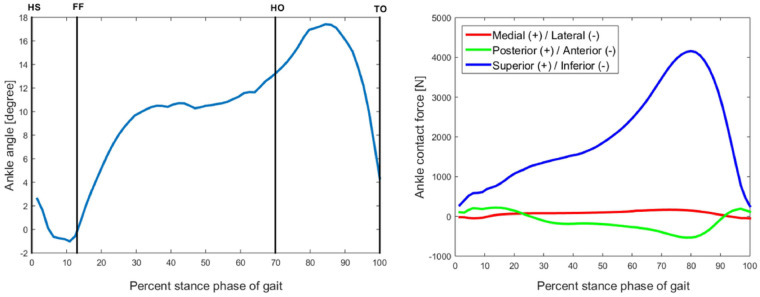
Kinematics of the ankle joint and applied ankle contact force. The force is applied on the talus. HS: heel strike (0% of stance); FF: foot flat (13% of stance); HO: heel off (70% of stance); TO: toe-off (100% of stance).

Gait data were input to a subject-specific MSK ankle model, built in NMSBuilder^41^ and tested for sensitivity.^42^ The OpenSim^43^ inverse kinematics (IK) tool was used to estimate the tibiotalar angles (Figure 1). Maximum marker errors were below 1 cm for all the considered frames.^44^ Inverse dynamics (ID) and static optimisation^45^ were run to estimate muscle forces. The joint reaction analysis tool^46^ was then used to estimate the joint force at the centre of the ankle joint (Figure 1 and Table 1). IK, ID and joint reaction analysis were performed every 0.01 s, subdividing the stance phase into 65 time points.

**Table 1.** Subset of the 65 ankle angles and ankle contact forces during the stance phase.

Percentage of stance	Ankle angle	Medial/lateral +/− (N)	Posterior/anterior +/− (N)	Superior/inferior +/− (N)
12	−0.57	13.64	21.7	72.5
20	5.37	65.25	143.87	1071
55	10.7	106.86	−22.14	2047
69	12.92	158.82	−35.45	3209.7
78	16.31	159.94	−52.52	4078.26
87	17.1	89.32	−33.88	3714.94
92	15.1	28.2	−29.46	2741.62

### Extended DEM implementation

EDEM, an extension of DEM^20,23,24,27^ was used to model the contact between talus and tibia. Talus and tibia were modelled as rigid, triangulated surfaces. More specifically, the articular regions of the right talus and tibia were identified and selected using MeshLab^38^ and Blender (https://www.blender.org/), discretised into 7617 triangular contact elements (average triangle area: 0.15 mm^2^) and imported into MATLAB (MathWorks, Natick, MA) to create a virtual alter ego of the real joint. The number of elements was chosen after a convergence analysis, with active contact area and peak contact pressure as metrics of convergence.

The anatomical reference frames constructed in the MSK model were used to set the joint angle. The tibia was rotated about the ankle axis according to the angles computed from IK (representative angles are reported in Table 1). This operation was performed for each of the 65 time points in which the stance was subdivided. After the rotation of the tibia, a mattress of springs was generated on the talar articular surface. Each spring had origin in the centre of a talar triangle and direction normal to it. Its second attachment point was determined by extending the normal until it intersected the articular surface of the tibia. The spring length was calculated as the distance between the two attachment points^24^ and updated at each time step, to account for the changes in relative pose of tibia and talus as the stance progressed. It was therefore neither homogeneous in space nor constant in time.

A threshold of 3.5 mm was set to discriminate whether a spring was representative of a contacting point or not. This value was chosen as it is twice the thickness of a typical undeformed cartilage layer in the tibiotalar joint.^47^ The nominal contact region was identified after removing from the computational domain the springs whose length was above the threshold.

**Figure 2.**
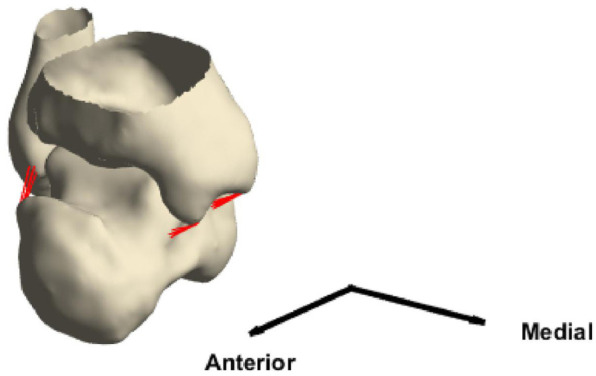
The geometries of tibia and talus connected by the ankle ligaments.

To make the model more anatomically consistent, four ligaments (anterior and posterior tibiotalar, anterior and posterior talofibular) whose attachment points were identified from the MRI were included as bundles of linear springs (Figure 2), with Young’s modulus set to 255 MPa.^48^

The joint contact force, computed from the MSK model, was applied as a point load at the centroid of the talus. The application of the load displaced the talus from its current position, while the tibia was fully constrained in the position prescribed by the kinematics. At each time point *t*, the attachment point of the *i*th spring on the talar surface translated by an amount $u_{t}^{i}$ with respect to its previous equilibrium position. The rotations of the talus were set to zero for all time points, so that it could only translate with respect to the tibia. Such displacement caused the spring, whose stiffness was $k_{t}^{i}$, to produce a force

(1) $$f_{t}^{i}=k_{t}^{i}u_{t}^{i}+f_{t-1}^{i}$$

where $f_{t-1}^{i}$ is the push-back force the spring is exerting because of its compressed state at time t−1. This was needed to ensure that the springs kept their compression state after a decrease of the applied joint contact force, therefore allowing a backward motion of the talus. The first term on the right hand side of equation (1) represents the increment of force due to the displacement of the talus from its previous position. The equilibrium of the system was ensured by imposing the balance of the total force produced by all the springs against the applied joint contact force.^20,23,24^

In order to calculate the stiffness of the spring at each time instant, we used the following estimation

(2) $$k_{t}^{i}=\frac{E\left(1-\nu \right)}{\left(1-2\nu \right)\left(1+\nu \right)}\frac{A^{i}}{h_{t}^{i}}$$

where $A^{i}$ is the area of the *i*th triangle on the talus and $h_{t}^{i}$ is the local cartilage thickness. Young’s modulus *E* and Poisson’s ratio ν, 10.35 MPa^20^ and 0.4247, respectively, were homogeneous over the joint.^23^

To comply with the requirement that contacting points are associated with springs in compression,^49^ non-compressed springs were removed from the nominal contact region at the considered time point. The equilibrium equation was then reformulated on the new domain and solved iteratively until only compressed springs were left. The resulting domain represented the current active contact region. Once the contact force on a spring was known, the contact pressure was computed dividing its normal component by the area of the triangle where the spring was located.

**Figure 3.**
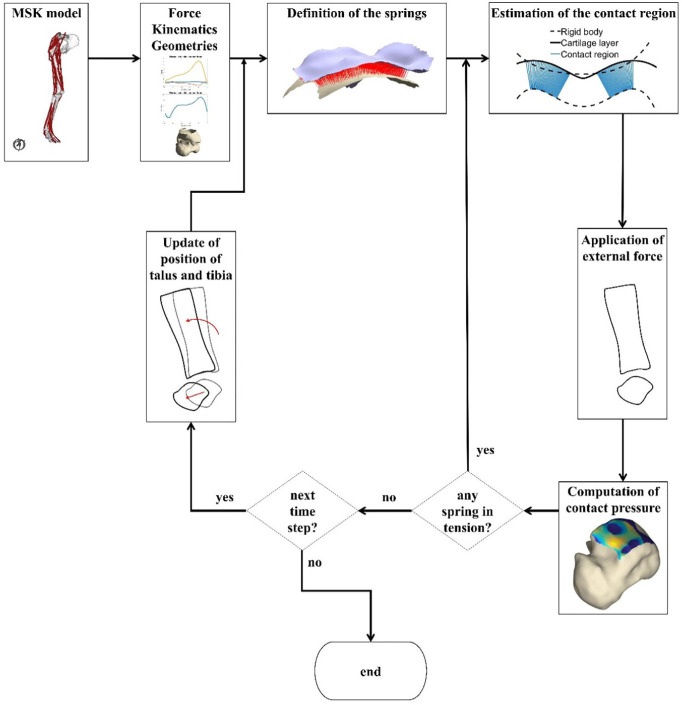
Schematic pipeline of MSK and EDEM. Ankle force, kinematics and the geometries of tibia and talus are input to the contact model. Compressive springs are defined over the articular region, providing the first estimate of the contact region. This estimate is then refined by removing the springs whose length is above a given threshold and by iteratively eliminating the stretched ones. After the algorithm has reached convergence, tibia and talus are oriented according to the measured kinematics and the next time point is simulated. In DEM, the stage ‘Update of position of talus and tibia’ is replaced by ‘Update of the position of tibia’.

Before proceeding to the next time step, the talus was moved according to the computed displacement, the stress state of the springs stored to be used in equation (1), and all the springs made again available for a possible contact engagement. The updated position of the talus was then used as initial position for the following time point. A decrease in the joint force would cause the talus to move backwards towards its original position and may reduce the extent to which the springs are compressed. The presence of the push-back force guarantees that the springs can experience some decompression before reaching a tensile state and being removed from the load bearing domain. The pipeline of the work is depicted in Figure 3.

### DEM implementation

DEM was implemented in a similar fashion as EDEM but with few key differences, the most important being the assumption that at each time point the talus was never displaced from its original position.^20,27^

The same geometry, reference frames and ankle axis of rotation as before were used. At each time point, the tibia was rotated with respect to the talus according to the orientation prescribed by the kinematics and, after this, a mattress of spring was placed on the talus to model the articular cartilage of the ankle joint. The length of each spring was computed, as in EDEM, at each time step. Since the talus was always located in its initial position, the push-back force was not needed, making the force exerted at time *t* by the *i*th spring

(3) $${\tilde{f}}_{t}^{i}=k_{t}^{i}{\tilde{u}}_{t}^{i}$$

The equilibrium equation which imposes the balance of the spring forces against the joint contact force was then solved iteratively with the requirement that only compressed springs were present. At each time point, the tibia was fully constrained to the position specified by the ankle kinematics and did not move during the search for the equilibrium configuration of the system. It is important to notice that in DEM the displacement of the talus, and consequently of the springs, was only used to compute the force expressed by the springs at the time point under examination but was not used to update the position of the bone. The displacement ${\tilde{u}}_{t}^{i}$ is therefore a displacement relative to the initial position of the *i*th point on the talar surface. After the solution at time *t* was found, the algorithm advanced to time t+1 by rotating the tibia according to the prescribed kinematic angle, while the talus was left in its original position.

### Sensitivity analysis

A global sensitivity analysis was run to assess the dependency of the EDEM outputs on two parameters determined from the literature: the average thickness of the undeformed cartilage layer,^47^ which was used to set the springs length threshold used to define the contact region, and the ligaments’ Young’s modulus.^48^ The effect of the cartilage Young’s modulus was not investigated, because under the adopted modelling assumptions it affected the contact pressure linearly. As equation (2) shows, Poisson’s ratio affects the contact pressure linearly with respect to the values of the function s(ν)=((1−ν)/((1−2ν)(1+v))). Furthermore, preliminary simulations showed that the contact area is only marginally affected by variations of ν. For this reason, and since the behaviour of s(ν) can be studied analytically, the analysis of the effect of Poisson’s ratio was not included in the sensitivity analysis.

We assessed the variation of peak contact pressure when the springs length threshold $h_{T}$ and Young’s modulus of the ligaments $E_{\mathrm{lig}}$ varied uniformly within the ranges [2.5, 4.5] mm and [200, 350] MPa, respectively. The parameter space was discretised into 20×20 points. Sensitivity to the inputs was assessed by evaluating the gradient of the peak pressure.

## Results

**Figure 4.**
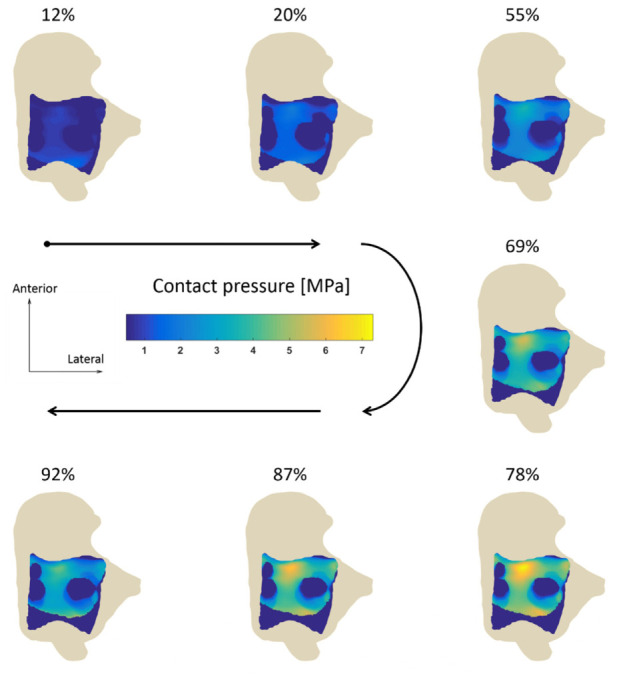
Pressure distribution on the talus at selected time points of the stance, computed using EDEM. The arrows indicate the progression of stance. The pressure increased as the talus was displaced towards the tibia, reaching its maximum at 78% of the stance phase, and then decreased as the talus was displaced backwards.

EDEM predicted that from the instant of the heel strike until the end of the mid-stance the talus was displaced superiorly, towards the tibia. At about 80% of the stance, before toe-off, the talus was located 0.57 mm superiorly with respect to its original configuration. Displacements in the medio-lateral and antero-posterior directions were substantially smaller, reaching maximum values of less than 0.1 mm. The decrease in the applied force after toe-off drove the talus back towards its original position. The active contact region evolved smoothly over time (Figure 4). In early stance, the loaded region was located on the posterior and then moved towards the anterior part of the talus as the gait progressed. At the same time, the active region became larger reaching its maximum extension before toe-off, sharply shrinking afterwards. The maximum value of contact pressure was reached at 80% of stance on the anterior part of the talus where, in this individual and at 16.9 degrees of dorsiflexion, the distance between tibia and talus was minimum. Contact pressure showed local peaks in the posterior and anterolateral parts of the talus as well. These three zones encircled a region, close to the centre of the articular surface of the talus, which was always inactive.

**Figure 5.**
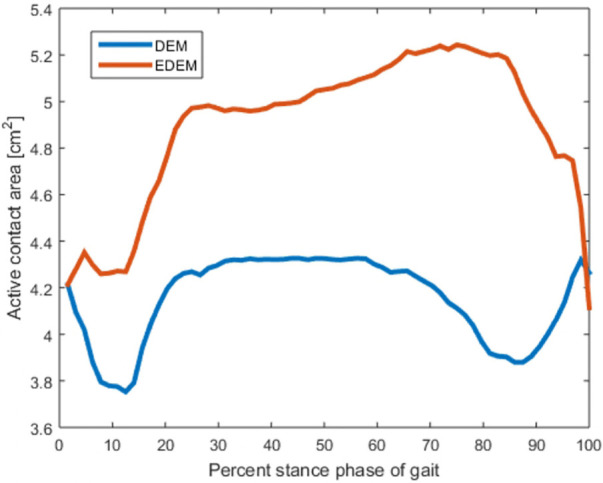
Active contact area during the stance, as computed by EDEM and DEM.

Despite similarities emerged in the contact patterns computed by EDEM and DEM, differences were present. EDEM predicted that a larger portion of the joint was involved in contact during stance: average active contact area was 4.67 cm^2^, whereas for DEM it was 4.18 cm^2^, 11% smaller (Figure 5).

**Figure 6.**
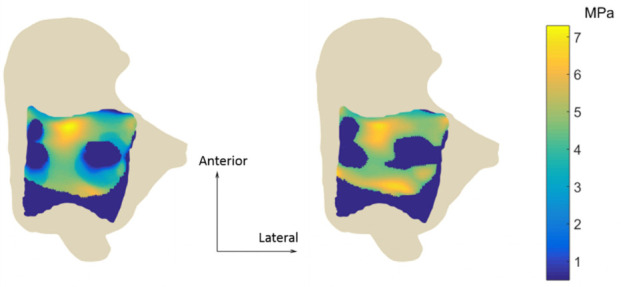
Pressure distribution at the instant of maximum loading (78% of the stance). EDEM is on the left, DEM on the right.

Both EDEM and DEM predicted the anterior part of the talus as the most loaded region, but the contact pressure from DEM was more evenly distributed over the load bearing domain, whereas EDEM predicted regions where the pressure was more concentrated (Figure 6). This held for every time point of the simulation. Maximum values were 7.3 MPa for EDEM and 6.92 for DEM.

**Figure 7.**
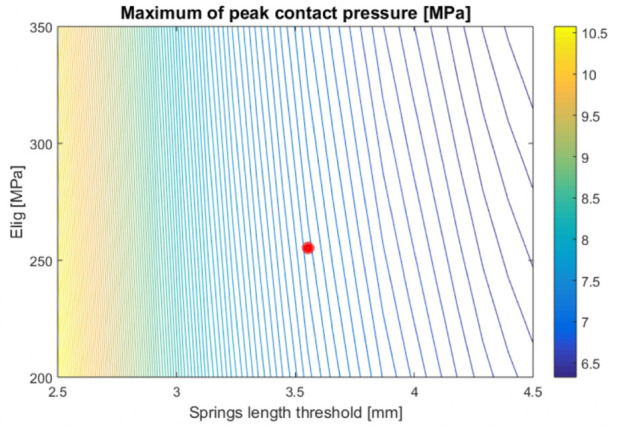
Dependency of the peak contact pressure on thickness threshold and Young’s modulus of the ligaments, computed using EDEM. The red dot indicates the nominal values used for the simulation of the full stance.

In the whole parameter space, the maximum value of the peak contact pressure was mostly influenced by the thickness threshold while the effect of Young’s modulus of the ligaments was less pronounced (Figure 7). As an example, if the threshold is kept constant to 3.5 mm, an increase of 175% of Young’s modulus of the ligaments from 200 to 350 MPa causes the maximum peak contact pressure to decrease by 3%. Conversely, keeping Young’s modulus fix to 255 MPa and increasing the threshold from 2.5 to 4.5 mm, one obtains a variation of 30% in the maximum peak contact pressure.

## Discussion

The aim of this study was the development of an extended formulation of DEM to provide a more functionally consistent estimation of the ankle contact pressure by considering the effects of the relative translation between talus and tibia on the determination of the contact pressure. Global measures reported in the literature, such as peak contact pressure or contact areas, tend to be homogeneous and easier to compare than local ones, such as the location of the most loaded region in the joint, which are scattered and allow only qualitative comparisons. Several authors^7,50–54^ have investigated the contact features of cadaveric ankles in different positions, using a large variety of applied load, geometries and experimental setups. In Calhoun et al.,^7^ the contact area was reported to increase as the ankle went from plantarflexion to dorsiflexion. Macko et al.^51^ observed similar results, with measured values ranging from 3.81 to 5.40 cm^2^, while some reported opposite behaviour.^55^ Kimizuka et al.^5^ applied loads ranging from 200 to 1500 N on eight cadaveric ankles obtaining contact areas from 1.96 to 6.18 cm^2^. In vivo imaging techniques were used by Wan et al.^11^ to measure the contact area during stance, observing values between 2.72 and 4 cm^2^. The ankle of the subject investigated in our study was in dorsiflexion for the majority of stance. The contact area increased when the dorsiflexion angle increased, with an average value of 4.67 cm^2^, in line with experimental results reported in the literature by Calhoun et al.^7^ and Siegler et al.^56^ in their experimental studies.

EDEM predicted two regions, at the centre of the talus, which were inactive during the whole simulation. In vivo^11^ and computational^23^ investigations of ankle contact mechanics have reported the presence of inactive regions, whose existence is strongly dependent on the subject-specific geometry of the individual under examination. In our model, each spring was assigned a length computed as the distance between the talus and the tibia, and springs whose length was above a maximum threshold were removed from the computational domain. On the tibial plafond of our subject, two depressions were present, which made the computed thickness higher than the threshold.

In Tochigi et al.,^54^ cadaveric ankles axially loaded with 600 N were subjected to 5 MPa of peak pressure, the most loaded region being in the anterolateral part of the joint. Similar results were obtained by Kimizuka et al.,^5^ with peak pressure of about 10 MPa. Conversely, Vrahas et al.^52^ observed concentration of contact pressure in the anteromedial part of their specimens. Peak values ranged from 1.9 to 12.4 MPa. In Suckel et al.,^9^ a dynamic study on eight cadaveric joint, the maximum pressure is reported to be located on the lateral side in half of the cases, and on the medial in the remaining, with average values about 4 MPa. In silico studies predicted maximum contact pressure of 3.74,^57^ 4,^58^ 8^59^ and 14 MPa^60^ and under a large variety of applied loads. Being the results from the literature obtained under many different conditions, only qualitative comparisons are possible. Maximum value of the predicted peak contact pressure (7.3 MPa) and its location, the anterior part of the talus, are aligned with findings reported in the literature.

Although EDEM and DEM predicted similar peak values, the active regions and the pressure distributions were different. The first step in estimating the contact region was, in both methods, the evaluation of the cartilage thickness and the elimination of the springs longer than a threshold. Pushed by the ankle contact force, in EDEM the talus moved towards the tibia. This resulted in the computed cartilage thickness being lower for EDEM than it was for DEM, allowing more springs to make contact. This increases the contact area and explains the greater engagement shown by EDEM in Figure 5. Despite the active area being larger in EDEM, we observed higher values of peak contact pressure and a less uniform pressure distribution. According to equation (2), strained springs exhibited higher stiffness and therefore created regions where the pressure peaked, leaving the remaining springs a minor share of load to hold. Peaks of cartilage strain were also observed by Wan et al.^11^ In DEM, the talus was never displaced from its initial position, causing the springs to have more uniform thickness and a more even pressure distribution. Some parts of the joints could be seen as not making contact when the ankle mechanics is investigated with single time point analysis, or when static methods which do not include the translations are used. However, since long-term contact stress exposure is thought to be associated with the propensity to cartilage degeneration,^60,61^ a less conservative estimate could be a precious tool for the early identification of the degeneration process.

The global sensitivity analysis showed that EDEM is more sensitive to the thickness of the cartilage than it is to the material properties of the ligaments. Modification of Young’s modulus of the ligaments had a limited effect on the peak contact pressure, which was reduced when the modulus increased. This is coherent with observations in the literature^62,63^ that the motion of the ankle is mostly determined by the topology of the articular cartilage. Increasing the threshold allows for more springs to be recruited for participating to the contact, explaining the strong effect this parameter had on the contact pressure.

This study has some limitations. First of all, despite the cartilage was modelled as strain dependent, we neglected its viscoelastic behaviour. On the other hand, the assumption of elastic behaviour is relatively common in the development of computational models of the joints,^20,25,64^ and it is generally regarded as appropriate in view of the loadings and time scales considered.^65^ Second, the EDEM/DEM modelling framework assumes that the bones are rigid and the contact kinematics is rigid too. Although computational studies on the hip have shown that modelling the bones as deformable affects the magnitude and pattern of the contact pressure,^12^ the assumption of rigid bones has been proved valid from studies on the knee^14,66^ and ankle.^57,67^ Third, the cartilage thickness was computed from MRI scans acquired in supine position, leading to estimated thickness greater than it would have been if the scans were taken with the subject standing. Also, the threshold for cartilage thickness was set using measures, gathered from the literature, on the thickness of an undeformed layer of cartilage. The inclusion of cartilage thickness data from the MRI scans would increase the computational complexity of the model, but also its resemblance to the anatomy of the subject. The quality of the acquired medical images, however, did not allow for a precise identification of the layer of cartilage covering the articulating bones. In addition, the adopted representation of the ligaments is simplified, and the results might benefit from a more detailed model of their behaviour; however, the limited effect of their stiffness in the sensitivity analysis confirmed that for physiological movements this is not critical.

Finally, a comprehensive validation against experimental results was not possible. Availability of fluoroscopy data, in conjunction with intra-articular pressure measurements, could provide an accurate quantification of the effect that bone translations have on the contact pressure, and prove the advantages of accounting for them in EDEM.

In conclusion, this article presented an extension of DEM capable of tracking the movement of the talus over time. Including the translation of the talus generated regions of higher cartilage stiffness, resulting in uneven stress distribution. Thus, difference between EDEM and DEM was more evident in the size and shape of the contact area than in the peak pressure values.
